# Health care workers’ knowledge and attitude towards TB patients under Direct Observation of Treatment in Plateau state Nigeria, 2011

**DOI:** 10.11694/pamj.supp.2014.18.1.3408

**Published:** 2014-07-21

**Authors:** Luka Mangveep Ibrahim, Idris Suleiman Hadjia, Patrick Nguku, Ndadilnasiya Endie Waziri, Moses Obiemen Akhimien, Phillip Patrobas, Peter Nsubuga

**Affiliations:** 1 Nigeria Field Epidemiology and Laboratory Training Programme, Nigeria; 2 Department of Community Medicine, Ahmadu Bello University, Zaria, Nigeria; 3 Tuberculosis Unit, World Health Organization Country Office, Abuja, Nigeria; 4 Global Public Health Solutions, Decatur GA, USA

**Keywords:** Tuberculosis, adherence, attitude, knowledge, health care workers, Nigeria

## Abstract

**Introduction:**

Tuberculosis (TB) is a public health problem in Nigeria. Adherence to the total duration of treatment is critical to cure the patients. We explored the knowledge of the health care workers on management of TB patients including their perceived reasons for patient non adherence to treatment to develop strategies to improve the quality of the TB control service in the state.

**Methods:**

We conducted a cross sectional study. We used self administered questionnaire to extract information from the health workers on their trainings for TB control, knowledge of the control services, patients’ education including prevention of defaulting from treatment. We conducted focus group discussion with the health care workers. We performed descriptive analysis using epiInfo software.

**Results:**

Of the 76 respondents 41 (53.9%) were female, 39.9% were community health extension workers, 26.3% were nurses/midwifes 30.3% lacked training on management of TB patient. Only 43.4% knew when to take action on patients who miss their drugs in the intensive phase, 30.3% and 35.5% knew defaults among category 1 and category 2 in the continuation phases of treatment respectively. They identified side effects of drugs (80%), daily clinic attendance (76.3%), health workers attitude (73.4%) and lack of knowledge on duration of treatment (71.1%) including their unfriendly attitudes towards the patients as the major barriers to patients’ adherence to treatment.

**Conclusion:**

Lack of knowledge of the health care workers on management of TB patients and poor interpersonal relation and communication with patients have negative effect on patients’ adherence to the long duration of TB treatment.

## Introduction

Tuberculosis (TB) is still a global public health problem two decades after it was declared a global emergency. Only 22 countries account for 80% of the TB burden in the world and Nigeria with estimated incidence of 133 cases per 100,000 populations is one of the highest in Africa [1]. The country uses the World Health Organization (WHO) recommended directly observed treatment short course (DOTS) strategy for the control of the disease with an expected target of at least 85% treatment success rates [2]. The achievement of the set targets is dependent on TB patients’ ability to adhere to the total duration of treatment prescribed by the DOTS strategy.

Under the DOTS strategy patients are classified into two categories, category 1 (Cat 1) are new patients or patients who had been exposed to anti TB drugs for less than 4 weeks and category 2 (Cat 2) are patients who had had previous exposure to anti TB drugs for at least 4 weeks [3]. A critical component of the strategy involves the direct observation of the patient swallowing their drug by a health care worker in the intensive phase of therapy for category 1 and during the entire duration of treatment for category 2 patients. The direct observation of the treatment is to ensure that patients adhere to the treatment to prevent default from treatment, failure of the treatment, and emergence of multi- drug resistant TB (MDR-TB) [4]. The onus for patients’ adherence to the treatment lies with the health care workers because they are the main source of information on TB to the patients. They remain in contact with the patient from diagnosis to the end of treatment. They have the strategic role to counsel and educate the patients including their families on the disease, its treatment and duration for the treatment. They are also responsible to ensuring access of the services to patients, promptly detect patients who miss their drugs and initiate the process for retrieval of the patient back to treatment [5].

TB patients on the other hand have the responsibility to follow the prescribed and agreed treatment plan and to conscientiously comply with the instructions given to protect the patient's health, and that of others. Furthermore, they have the responsibility to inform the health providers of any difficulties or problems with following the treatment or of any part of the treatment as contained in the patients charter for TB care [6]. The complex relationship between the patient and health care workers is an important determinant of the outcome of TB treatment. Positive relationship or interaction will lead to good outcome of treatment and vice versa. The success of the interaction is affected by the knowledge of the health care workers on the disease and treatment protocol, their skills on patients counselling and education and their attitude towards the patients. Patients who are poorly counseled or educated on TB and its treatment may end up with poor outcomes; similarly a negative attitude of the health care workers towards the patients will cause them to stop the treatment.

In Plateau state, TB control using the DOTS strategy started in 2001 with only five treatment centers but by the end of 2011 there were 198 health facilities where TB patients can access treatment. These centers included public, private for non for profit (faith based), and private for profit facilities. Despite the decentralization of TB treatment centers, review of treatment outcomes from 2001 to 2010 showed that Plateau state the highest cured ever achieved was 63.5% in 2006 this is far lower than the expected targets of at least 85% and the lowest default rate of 6.5% was higher than the expected target of less than 3% set by the National control program [7]. The contribution of the health care workers knowledge on TB and its management including their attitude towards patients on the outcome of treatment is not known in the state. Additionally, no study had been done on the knowledge of the health care workers on TB and management of TB patients including their attitude towards the patient in the state. Exploring the health care workers’ factors associated with adherence to TB treatment in the state will help the control program to identify and address the gap for effective management of the TB patients to improve on the quality of the TB service in the state.

## Methods

### Study setting

The study was conducted in Plateau state; it is one of the states in the north central geopolitical zone of Nigeria with a population of 3.8 million people. We conducted a cross-sectional study between June and July 2011 among health care workers serving as TB (DOTS) focal persons or participating in the TB control services in the health facility. The health care workers included medical doctors, nurses, midwifes, community health extension workers, junior community health extension workers and environmental health officers.

### Sample size, data collection and analysis

The health facilities providing TB control services were used to select the health care workers. There were 198 DOTS facilities in the state comprising 174 public, 19 faith-based (private for non-profit) and 7 private for profit facilities. One quarter (25%) of the health facilities were included in the study because of limited time and resources. Making provision for 10% non responses gave a minimum sample size of 55 health care facilities.

A proportionate to size allocation was used to determine the number of facilities from the various categories of health facilities as follows: Px55/198 where P was the type of health facilities; Public health facilities = 172x55/198 = 48; Faith Base facilities = 19x55/198 = 5; Private for profit = 7x55/198 = 2

The facilities selected included 48 public facilities, 5 faith base facilities and 2 private for profit facilities. The lists of all the health facilities offering TB control (DOTS) services were obtained from the state TB control office. The facilities were listed by their types to make up the sample frame; each facility in each group was assigned serial numbers. Using the number from the predetermined weighted sample size calculation the facilities were selected using systematic sampling method. All the health care workers involved in the TB control services who were on duty on the day of visit to the selected facility were involved in the study. The health care workers were interviewed using self administered structured questionnaire on their socio-demographic characteristics, type of training received for TB control services, including knowledge on TB control services, patients’ management and factors associated with outcome of treatment.

We conducted focus group discussions (FGDs) with homogeneous groups among the selected health care workers. Using a pre-designed FGD guide we asked questions on knowledge and factors responsible for poor outcome of TB treatment. We entered the data into an Epi Info version 3.3.2 database and we performed descriptive analysis. The knowledge of the health care workers on TB control was scored based on the number of points mentioned by the health workers into poor, fair and good knowledge of the subject matter. We developed and used a 5-point domain to grade knowledge, for each domain; one point scored for correct response, zero for wrong response. The knowledge grade was as follows; a) 0 to 1 points: poor knowledge, b) 2 to 3 points: fair knowledge and c) 4 and above good knowledge Qualitative data from the FGDs were transcribed into written form and analyzed according to the specific themes.

### Ethical consideration

We obtained ethical clearance for the study from the Plateau state ethical review committee. Informed consent was also obtained from all respondents involved in the study.

## Results

**Figure 1:**
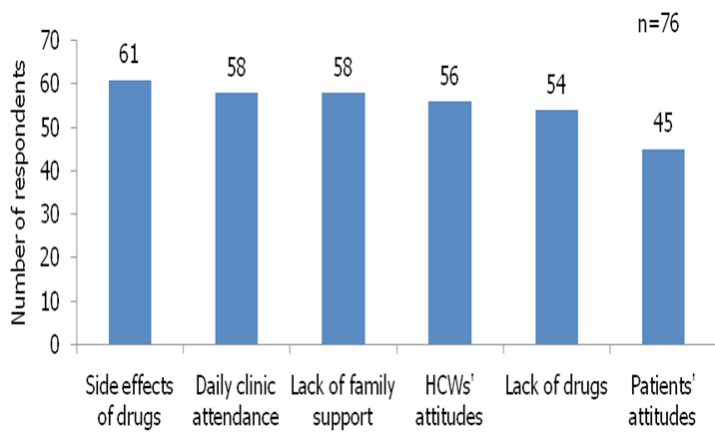
Health care workers perceived causes of patients’ non adherent to TB treatment

A total of 76 health care workers were interviewed, 41 (53.9%) of them were female. Forty percent were community health extension workers, 26.3% were nurses or midwife, and medical doctors accounted for 15.8%. Majority (82.9%) of the health care workers had been working in their respective health facility for more than one year (Table 1) and 30.3% of the health care workers had never received any form of training on TB control services in the state. On their knowledge of treatment default and when to take action for retrieval, only 43.4% knew when to take action on patients who miss their drugs in the intensive phase, 30.3% and 35.5% knew defaults among category 1 and category 2 in the continuation phases of treatment respectively (Table 2). On their perceived reasons for patient non adherence to treatment 61 (80%) mentioned side effects of drugs while daily clinic attendance, health workers attitude, lack of knowledge on duration of treat, and patient attitude was identified by 58 (76.3), 56 (73.4), 54 (71.1%), and 45 (59.2%) respectively (fig. 1).

**Table 1:** Socio-demographic characteristics of Health care workers

Characteristics of Health workers (n = 76)		Frequency	%
**Sex distributions**	Female	41	53.9
	Male	35	46.1
**Highest education qualification**	Medical Doctor	12	15.8
	Nurse/Midwife	20	26.3
	Community health officer	4	5.3
	Community health extension worker	25	32.9
	Junior community health extension worker	7	9.2
	Environmental health officer	3	3.9
	Others	5	6.5
**Duration working in present health facility**	< 6 months	4	5.3
	6-12 months	9	11.8
	> 12 months	63	82.9

**Table 2:** Health workers’ knowledge of defaults among the clinical categories of patients

Health workers’ knowledge (n = 76)		Frequency	Percent
Default of TB patients in intensive phase	Correct response	33	43.4
	Wrong response	43	56.6
Default of Cat 1 in continuation phase	Correct responses	23	30.3
	Wrong responses	53	69.7
Default of Cat 2 in continuation phase	Correct responses	27	35.5
	Wrong responses	49	64.5

Majority of the health care workers had poor to fair knowledge on the concept of direct observation of treatment, the key educational messages to patient at registration for treatment, during treatment and on how to avoid default from treatment (Table 3).

**Table 3:** Health care workers’ knowledge DOT and education messages

Knowledge for TB control services	Poor N (%)	Fair N (%)	Good N (%)	Total
Concept of direct observation of TB treatment (DOT)	18 (23.7)	26 (34.2)	32 (42.1)	76
Types of education messages at registration of patient	21 (27.6)	33 (43.4)	22 (28.9)	76
Types of education message to patient during treatment	28 (30.8)	30 (31.5)	18 (27.7)	76
What to do to avoid patients defaulting from treatment	31 (40.8)	23 (30.3)	22 (28.9)	76

Majority of the health care workers who participated at the FGDs knew that the current treatment for TB is the direct observation of the treatment by a health care worker and its importance in outcomes of treatment. Majority also knew the key education messages to the patient to ensure adherence to treatment.

Their perceived reasons for patients defaulting from treatment to include: the patient feeling better or well, the patients who experienced side effect of drugs, daily clinic attendance to collect drugs is too cumbersome to the patient especially patient who have to travel long distance and poor patient education by the health workers on the treatment. A female participant said *“I think some patients after taking the drug for 2 or 3 months they feel strong and so do not want to continue with the drugs. They will feel that they are well so no need to continue taking the drugs”* another female participant also said *“Getting well and disappearance of symptoms is major cause of interruption or default from treatment also the inconvenience caused by the side effects of drugs”*.

There was a general consensus among the participants that the health workers contribute significantly to patient adherence or non adherence to TB treatment. According to the participants, the major factors that are required for patient to adhere to their treatment were centered on patient-health workers relationships, patients’ education and proximity of health facility to the patient. The following quotations from members were noted as follows; *“The attitude of the health workers is very important; an unfriendly attitude will chase the patient away. The health worker should be friendly with the patients because if we are not friendly with them each time they come to take their drugs they will not be free”* (female participants).

*“Honestly I have ever heard some patients say “this nurse is too harsh, I do not want her to touch me” they become selective. So if you are a TB service provider and you do not show them love and concern, patients will just leave the treatment even though they know that they treatment is for their good because they cannot tolerate the bad attitude of the health worker”* (male participant).

*“If a patient interrupts treatment and we fail to trace the patient promptly, we should be blamed for the failure”* (male participant).

*“If we register patients to take treatment in a facility that is far from their homes they will not be consistent on their treatment”* (male participant).

They also identified the following as major barriers to effective education of patients: a) lack of knowledge of the health workers on TB and its treatment, b) lack of communication skills by the health workers and c) health workers’ unfriendly attitude towards the patient.

## Discussion

Our study shows that outcome of TB treatment in Plateau state is affected by the knowledge of the health care workers on the disease, its treatment protocol, and the attitude of the health care towards the patients. Patients’ decision to stay on treatment for the prescribed duration depends on how much help they get from the health care workers which also depends on the knowledge of the health care workers. The disappearance of the disease even within few weeks of initiation of therapy might make the ill informed patient to feel that they are cured and thus stop their treatment. Counseling and education of the patient is a critical factor for the success of the treatment. The positive effect of TB patients counseling and education on the disease and treatment had been reported in Asia and Africa [8–10]. The researchers showed that proper adherence counseling to patients led to significant reduction in default rates among the patients. Similarly, treatment takes a long duration only patient were proper counseled by the health care worker would continue to take the treatment even their symptoms disappears or they fell well even after few weeks of initiation of treatment.

The ability of the health care workers to educate, counsel and even communicate well with the patients has bearing on their knowledge of the disease, patients’ management and the control strategies. This is dependent on the training they received on the TB control services. The health care workers without training on the control services will not be able to counsel the patient properly and will have negative effect on the patient adherence to treatment. We found in our study that though majority (82.9%) of our respondents had worked in facilities with TB services for more than 12 months, about a third of them had not benefited from either on-the-job training or workshop on the TB control services. The low knowledge score of the health care workers on TB control services might had been due to lack of trainings. Furthermore, health care workers with paucity of knowledge on the management of the TB patient under the DOTS will not be able to offer effective counseling to the patients [11]. It is also common place for patients to hold erroneous belief about their disease and treatment [12], to break such belief the health care workers must be equipped with the knowledge to communicate to the patient. This was corroborated by the health care workers who participated in the focus group discussions. They identified a major barrier to patients’ adherence to treatment to be improper education of the patients by the health care workers on the duration of treatment. the importance of training in the management of TB patient was noted by Mesfin et al in a study on quality of care among TB patients in Ethiopia they noted that patient who were managed by health care workers who were not trained were more likely to interrupt their treatment [13]. These findings might be the driving force behind the poor performance of the TB control services in Plateau state.

The relationship between the health care workers and the patients are critical elements in the success of TB treatment. The patients are expected to follow the instructions of the health care workers on their treatment; however, whether or not the patients follow the instructions depend on the attitudes of the health care workers adjudged to be negative or friendly by the patients. Negative attitudes will make patient lose confidence in the health care workers or they may feel threatened, not loved, or respected they may choose not to continue their treatment leading to interruption and eventually failure or default from the treatment. Many studies have noted the effect poor health care workers and patients relationships on TB patients’ adherence to treatment [14, 15], they reported that patients blamed the health care workers attitude them as reasons for defaulting from treatment. Patients who feel that they are not treated with respect and empathy including those who feel the health care workers was rude and unhelpful to them were unlikely to complete their treatment. Our respondents had similar views; they identified negative attitude of the health care workers towards the patients were a key barrier to patients’ adherence to treatment.

Our study had the following limitations; first we did not explore from the respondents the training they received in their places of studies on TB control services. Secondly we did not explore other factors such as work environment, tools and remuneration that can contribute to performance and attitude towards their works. Lastly we did not explore the non-formally trained sector such as the traditional healers who are contributing significantly in the health care delivery services especially in rural areas.

## Conclusion

The result of our study show that health care workers participating in TB control services in the state had poor knowledge on management of TB patients due to lack of training and negative attitude of the health care workers towards the patients is a potential barrier to patients adherence to treatment. Base on our findings we recommend that the Plateau state TB control program should conduct refreshers training for all TB focal persons, the training should include interpersonal communication and adherence counseling to all TB patients. The Plateau state TB and leprosy control program in collaboration with the coordinators of the primary health care at the LGAs level should carry support research on factors that affect health care attitude towards their works.
